# Germ–Somatic Cell Interactions Are Involved in Establishing the Follicle Reserve in Mammals

**DOI:** 10.3389/fcell.2021.674137

**Published:** 2021-06-14

**Authors:** Patrícia Rodrigues, Darlene Limback, Lynda McGinnis, Mónica Marques, Juan Aibar, Carlos E. Plancha

**Affiliations:** ^1^Centro Médico de Assistência à Reprodução (CEMEARE), Lisbon, Portugal; ^2^Escola de Psicologia e Ciências da Vida, Universidade Lusófona de Humanidades e Tecnologias, Lisbon, Portugal; ^3^Department of Pathology and Laboratory Medicine, University of Kansas Medical Center, Kansas, KS, United States; ^4^Department of Obstetrics and Gynecology, Keck School of Medicine, University of Southern California, Los Angeles, CA, United States; ^5^Instituto de Histologia e Biologia do Desenvolvimento, Faculdade de Medicina, Universidade de Lisboa, Lisbon, Portugal

**Keywords:** primordial follicle, follicle reserve, germ–somatic cells interaction, germ cell factor, somatic cell factor

## Abstract

Mammalian females are born with a finite reserve of ovarian follicles, the functional units of the ovary. Building an ovarian follicle involves a complex interaction between multiple cell types, of which the oocyte germ cell and the somatic granulosa cells play a major role. Germ–somatic cell interactions are modulated by factors of different cell origins that influence ovarian development. In early development, failure in correct germ–somatic cell communication can cause abnormalities in ovarian development. These abnormalities can lead to deficient oocyte differentiation, to a diminished ovarian follicle reserve, and consequently to early loss of fertility. However, oocyte–granulosa cell communication is also extremely important for the acquisition of oocyte competence until ovulation. In this paper, we will visit the establishment of follicle reserve, with particular emphasis in germ–somatic cell interactions, and their importance for human fertility.

## Introduction

Mammalian females have a finite reproductive reserve of oocytes, established during fetal life (human) or perinatally (mouse). This reserve is composed of a large number of primordial follicles (PF), in which the oocyte is enclosed by a small number of somatic cells—pre-granulosa cells (Rodrigues et al., 2009; Clarke, 2017). It is during PF formation that the ovarian follicle reserve is established (Pepling and Spradling, 2001; Rodrigues et al., 2009). The oocyte is the essential component of female reproduction and it must undergo growth and development within the environment of an ovarian follicle (Chang et al., 2017). This environment includes bidirectional communication that is absolutely critical for survival of the oocyte and the companion granulosa cells. The oocyte receives nutrients, metabolites, and extrafollicular signals from the surrounding granulosa cells directly influencing its differentiation. The oocyte also signals to the surrounding granulosa cells, inducing their proliferation and survival. This reciprocal communication between oocyte and somatic cells within the follicle is essential to support full oocyte development (Li and Albertini, 2013; Coticchio et al., 2015; McGinnis et al., 2018; review Wang et al., 2017).

Across reproductive life, the initial PF reserve gradually diminishes until it is exhausted, over months (mouse) and decades (human) of attrition (Wang et al., 2017; McGinnis et al., 2018). Unfortunately, 1% of women worldwide suffer early loss of their reserve, a condition termed premature ovarian insufficiency (POI), in which the PF reserve is exhausted before women reach 40 years old (Grive and Freiman, 2015). It has been suggested that POI may be due to a combination of multiple genetic mutations (Bouilly et al., 2015). Therefore, understanding how the follicle reserve is established may shed light on the female reproductive lifespan.

This review will focus on ovarian follicle assembly with particular emphasis on somatic–germ cell communication and its role in establishment of the follicle reserve and, consequently, mammalian female fertility.

## Ovarian Follicle Assembly

**FIGURE 1:**
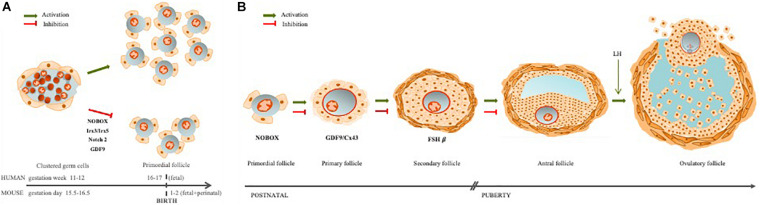
Ovarian follicle assembly and folliculogenesis. **(A)** The ovarian follicles are regulated by germ–somatic cell interaction. When this communication is disrupted, follicle assembly is impaired, leading to early follicle reserve loss (Irx3/5; Nobox, Notch2) or inability to acquire meiotic competence (GDF9). **(B)** Folliculogenesis occurs with oocyte growth, mainly due to a bidirectional communication between the oocyte and granulosa cells through TZPs. If this communication is disrupted, follicle development may arrest at different stages: primordial (Nobox), primary (GDF9/Cx43), or secondary (FSHβ).

In the mouse model, traditional views hold that oogonia proliferate within a cluster, through a series of incomplete mitosis, due to the speed of cell divisions (Pepling and Spradling, 1998; Rodrigues et al., 2009). These clusters are surrounded by mesonephros-derived somatic cells that form the ovigerous or rete cords, continuous with the surface epithelium of the ovary (Byskov, 1986; McLaren, 2003; Guigon and Magre, 2006). Within the ovigerous cords, at around fetal 15.5 in mice, and 11–12 weeks gestation in humans, oogonia enter into prophase of the first meiotic division and arrest in diplotene stage (van den Hurk and Zhao, 2005; Jones and Pepling, 2013; Grive and Freiman, 2015). Follicular histogenesis is initiated through a process of fragmentation of the ovigerous cords, alignment of epithelial pre-granulosa cells to surround individual primordial oocytes, and the attraction of mesenchymal cells to the follicle’s nascent basement membrane (van den Hurk and Zhao, 2005; Guigon and Magre, 2006). This coordinated interaction, between these different cell types, results in the formation of the lifetime pool of PF, known as the ovarian follicular reserve (Figure 1A).

This period of PF assembly, and establishment of the follicular reserve is associated with a significant germ cell loss, mostly by apoptosis (Rodrigues et al., 2009; Wang et al., 2020). However, other mechanisms of germ cell loss have been implicated in this process, like autophagy and oocyte shedding through the nascent ovarian epithelium (Rodrigues et al., 2009). Follicle assembly occurs during human’s fetal life, while in the mouse, follicle assembly occurs postnatally (Guigon and Magre, 2006).

The process of cluster breakdown starts in the medullary region of the ovary and progresses to the cortical area with follicle growth (Wang et al., 2017). This movement is supported by two different waves of pre-granulosa cell activation, giving rise to two different classes of PF (Mork et al., 2012; Zheng et al., 2014a). The first wave of pre-granulosa cells contributes to the formation of the first class of PFs in the medulla area and will be the follicles contributing to the onset of puberty (Zheng et al., 2014a, b; Wang et al., 2017), whereas the second wave of differentiating pre-granulosa cells contributes to the formation of the PFs involved in the second class of follicles that will start to grow within the ovarian cortex and support the female reproductive life in its end (Zheng et al., 2014a, b; Wang et al., 2017). Using single-cell RNA sequencing during PF assembly, at E16.5 (mouse) pre-granulosa cell differentiation and second wave initiation, an increase in the expression of stem cell markers *Wnt4* and *Aldh1a1* was detected, which are essential for retinoic acid biosynthesis (key regulator of meiosis) (Zhang et al., 2018; Wang et al., 2020). Interestingly, it was suggested that instead of meiosis entry, the geographic location of the PF within the ovary may have a greater influence in determining which PF will activate first (Cordeiro et al., 2015). Through design of a triple transgenic mouse for MVH/GDF9/ZP3, the authors observed that the ventral side of the ovary has an earlier onset of meiosis, but the first PF formed and activated were located in the dorsal region (Cordeiro et al., 2015).

Interestingly, two members of the Iroquois homeobox transcription factor gene family (involved in patterning and embryogenesis), Irx3 and Irx5, are fundamental for correct PF formation (Fu et al., 2018). Deleting both factors in mice caused defects in both oocyte and granulosa cells during primordial follicle assembly (Fu et al., 2018). These results show that communication between all the cells involved is essential for proper germ cell cluster breakdown and PF assembly (Fu et al., 2018; Wang et al., 2020).

## Germ–Somatic Cell Interaction

Cell-to-cell communication is an important driving force during mammalian embryonic development, inducing cell movement, rearrangement, shape changing, etc., of which the ovarian follicle assembly is no exception (Wang et al., 2017). The bidirectional communication between germ cells and somatic cells is fundamental for follicle assembly and adequate follicle environment for acquisition of oocyte competence (Li and Albertini, 2013; Zhang et al., 2018).

The interactions between germ cells (oogonia and oocytes) and somatic cells (granulosa, theca, and stromal cells), vasculature, and extracellular matrix (ECM) are all essential components enabling follicle formation, growth, ovulation, and luteinization. Direct communication between an oocyte and its surrounding companion granulosa cells requires transzonal projections (TZPs) that extend from the granulosa cells closest to the oocyte (corona radiata cells), through the zona pellucida, and reaching all the way to the surface of the oocyte (Albertini et al., 2001; Combelles et al., 2004; review Li and Albertini, 2013). TZPs, containing actin filaments and/or microtubules (Li and Albertini, 2013), connect with the oocyte membrane (oolemma) *via* intercellular gap junctions and adherens junctions. Thus, the TZPs with open gap junctions provide the basis for germ–somatic cell crosstalk (Li and Albertini, 2013; Clarke, 2017; Wang et al., 2020).

Gap junctions are formed by groups of connexin proteins that interlock to form a pore in the cell membrane. The size of the molecules that pass through is regulated by the size of the pore, which depends on the specific gap junctions involved. The primary connexin found in granulosa cells is CX43 (GJA1), although others are also present (Simon et al., 2006; Wang et al., 2020). The primary connexin in the oocyte is CX37 (GJA4) (Simon et al., 1997). The two types of connexins form heterodimeric gap junctions between the TZPs and the oolemma, enabling the necessary cross-communication and passage of critical molecules that support development and survival of both the oocyte and the granulosa cells. Since Cx37 forms one of the smallest pores in the gap junction family, it is the oocyte that regulates the size of molecules that can pass between these two cell types.

**FIGURE 2:**
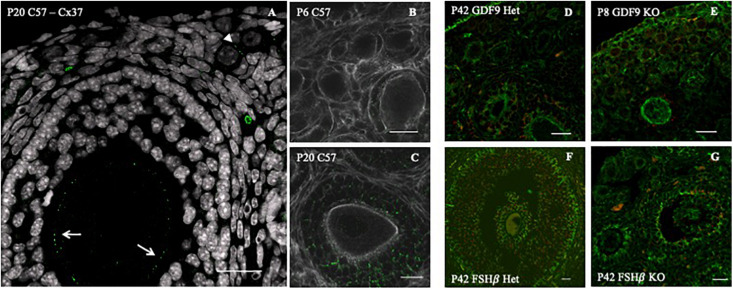
Connexin 43 cell–cell communication regulation in GDF9 and FSHβ knockout mice model. **(A)** Connexin 37 expression (green dots) in the oocyte of a secondary (white arrows) and primordial (white arrowhead) follicle of a P20 C57 female mouse ovary. Connexin 43 (green dots) distribution in C57 6 days post-birth (P6) **(B)** and 42 days postnatal (P42) **(C)**, double stained for actin (white). Connexin 43 (red) distribution with double staining for tubulin (green) in GDF9 heterozygous P6 **(D)** and homozygous **(E)** female. Connexin 43 (red) distribution with double staining for tubulin (green) in FSHβ P42, in heterozygous **(F)** and homozygous **(G)** female. Bars = 20 μm. Image adapted from Rodrigues (2016).

Previously, we observed that Cx37 and Cx43 are present in all follicle stages, including primordial (Rodrigues, 2016; Figures 2A–C). We found Cx43 to be impaired in GDF9 and FSHβ knockout mice [Rodrigues, 2016; Figures 2D,E (GDF9) and Figures 2F,G (FSHβ)]. Moreover, in the absence of Cx37 in female mice, the primordial germ cells fail to achieve meiotic competence. In the adult, there is no formation of ovulatory follicles (Simon et al., 1997, 2006), which reinforces the importance of gap junctions in normal ovary function. TZPs are impaired and cell–cell communication is compromised when both Irx3 and Irx5 are disrupted, leading to POI in mice (Fu et al., 2018).

### Oocyte Factors

Most of what is known today in mammalian ovarian development results from morphological descriptions of ovaries at different ages in various models and targeted disruption of genes in the mouse models (Roy and Matzuk, 2006). Transgenic animal models have enabled researchers to identify the regulators of ovarian function and to better understand human disorders, in particular those affecting reproduction.

The transforming growth factor β (TGF-β) super family, which, during development, mediates several cell behaviors, including proliferation, differentiation, ECM production, and cell death, includes some members implicated in follicle growth (Pangas and Matzuk, 2004; Sanfins et al., 2018). One member, Growth differentiation factor 9 (GDF9), is a germ cell-specific factor previously identified in growing oocytes (McGrath et al., 1995; Elvin et al., 1999a). The importance of this growth factor during early folliculogenesis was first demonstrated when Dong et al. (1996) inactivated the mouse GDF9 gene. Female mice lacking a functional GDF9 gene are infertile due to the arrest of folliculogenesis at the primary follicle stage (Dong et al., 1996; Elvin et al., 1999a, b; Sanfins et al., 2018; Figures 2D,E). Interestingly, Wang and Roy (2006) discovered that GDF9 was essential for the formation of PF in the hamster. They also found that FSH indirectly regulates oocyte GDF9 gene expression, implying that FSH regulates oocyte gene expression and primordial follicle formation. Recently, GDF9 expression in oocytes at all stages of development including primordial oocytes was confirmed through single-cell RNA sequencing (Wang et al., 2020).

Bone morphogenic protein 15 (BMP15) is another member of the TGF-β gene family. It is an X-linked gene, encoding an oocyte-specific protein (Dube et al., 1998; Sanfins et al., 2018). BMP15 is expressed in oocytes from primary follicles onward (Dube et al., 1998). It is very closely related with GDF9, in both protein structure and gene expression (Yan et al., 2001). In fact, double mutant mice (BMP15^–/–^GDF9^–/–^) show early oocyte loss, whereas BMP15 null females are sub-fertile (Yan et al., 2001). The synergy between both factors was demonstrated by improving oocyte quality and granulosa cell regulation with *in vitro* produced heterodimers GDF9-BMP15 (cumulin; Peng et al., 2013). BMP15 is also involved in regulating gene expression of Cx43, in human granulosa cells (Chang et al., 2017; Sanfins et al., 2018).

Another oocyte factor, involved in the germ–somatic cell communication is Nobox (Newborn Ovary Homeobox), an oocyte-specific homeobox gene expressed early in development within germ cell clusters, as well as primordial and growing follicles (Suzumori et al., 2002; Rajkovic et al., 2004). In the absence of Nobox, there is an accelerated postnatal oocyte loss (Suzumori et al., 2002). Mutations in the Nobox gene are associated with POI, as well as a delay in germ cell cluster breakdown (Rajkovic et al., 2004; Qin et al., 2007; Lei et al., 2010; Bouilly et al., 2015; Figure 1A).

Although there are more contributors, these oocyte factors are examples of the central importance of oocyte in the bidirectional communication necessary to establish an adequate reserve of ovarian follicles. Most importantly, they illustrate that it is not just one factor, or one cell type involved, but rather a combination of factors and communicating cell types.

### Somatic Cell Factors

Germ cell cluster breakdown is a well-coordinated process and one that demands intercommunication between germ and pre-granulosa cells for efficient PF formation and, with it, establishment of the ovarian follicle reserve (Wang et al., 2020). For example, when Notch 2 is conditionally mutated in granulosa cells, multi-oocyte follicles are formed, thus indicating an impaired germ cluster breakdown. The Notch pathway is an important signaling pathway during embryo development and Notch2 has been reported in both pre-granulosa and granulosa cells (Xu and Gridley, 2013; Wang et al., 2017; Figure 1A). Furthermore, a model of the conditional granulosa cell mutant for Notch2 also showed diminished oocyte apoptosis, increased oocyte survival, but significantly fewer normal PF due to the formation of multi-oocyte follicles (Xu and Gridley, 2013).

As mentioned above, important structures involved in germ–somatic cell interactions are the TZPs, which form gap junctions between the oolemma and the tip of the TPZ extended from the granulosa cell (Simon et al., 2006; Li and Albertini, 2013). The inability to produce pups in the absence of intracellular communication between germ and somatic cells in Cx37 null female mice (Simon et al., 1997), as well as the follicular arrest at primary follicle stage in Cx43 null females (Simon et al., 2006) are significant demonstrations of the importance of this cell–cell communication.

As mentioned previously, loss of IRx3 and IRx5 in female mice caused defects in granulosa cells, particularly in the mis-location of gap junction proteins, resulting in a premature oocyte loss (Fu et al., 2018). Interestingly, Irx5 expression was observed exclusively in granulosa cells while Irx3 alternated between the granulosa and the oocyte during PF assembly (Fu et al., 2018).

## Other Factors in Ovarian Follicle Development

It is well known that adult ovary events, such as antral follicle growth, maturation, ovulation, and luteinization are primarily controlled by two gonadotropins: follicle stimulating hormone (FSH) and luteinizing hormone (LH), secreted by the pituitary gland. In turn, the pituitary is controlled by gonadotropin-releasing hormone (GnRH), produced in the hypothalamus (Gougeon, 1996; McGee and Hsueh, 2000). Interestingly, Cx43 expression in granulosa cell is hormonally regulated by both gonadotrophins (FSH and LH) (Granot and Dekel, 1998; Chang et al., 2017; Figures 2A,B,F,G).

FSH receptors (FSHr) are transmembrane G-protein-coupled receptors that localize in granulosa cells in the ovary (Kumar et al., 1997, 1999; Oktay et al., 1997; Burns et al., 2001). Primordial follicle activation and the first phases of follicle development are independent of FSH, as evidenced by the presence of preantral follicles in FSH receptor-null and FSHβ-null female mice (Danilovich et al., 2000; Kumar, 2005). Apparently, loss of FSH receptor or FSHβ did not prevent primordial follicle formation or pre-antral follicle development; however, follicular development was arrested at the preantral stage (Kumar, 2005). There is contradictory evidence for the role of FSH in formation of PF. As mentioned earlier, Wang and Roy (2006) found that FSH activity was essential to PF formation in the hamster, and more recently, it was reported that FSH promotes PF formation in mice through activin stimulation (Lei et al., 2010). This suggests the possibility that other proteins or signaling pathways can compensate for loss of FSH, depending on the research model. Interestingly, 17β-estradiol (E_2_), which regulates FSH action, was found to inhibit cluster breakdown when added to mouse neonatal ovary cultures (Grive and Freiman, 2015).

Together, oocyte and somatic factors contribute to ovarian follicle reserve establishment and demonstrate how this is a process of cooperation between different cell types within the ovary.

## Follicle Reserve and Acquisition of Oocyte Competence

Like PF formation and ovarian follicular reserve assembly, intercellular communication is fundamental for acquisition of oocyte competence (Coticchio et al., 2015; McGinnis et al., 2018).

Briefly, the ovarian follicle reserve is established early in development, and the primordial oocyte, arrested in prophase I of meiosis I, can stay dormant for most of female fertile life. However, periodically, some PFs are recruited to start growing. This follicle growth also initiates oocyte hypertrophy, for storage of macromolecules and organelles in oocyte cytoplasm in order to sustain the first embryonic developmental stages (Li and Albertini, 2013; Clarke, 2017; McGinnis et al., 2018). Following puberty, it is from the pool of growing follicles that some follicles are periodically selected to ovulate a mature oocyte (Coticchio et al., 2015; McGinnis et al., 2018). Communication between oocyte and granulosa cells continues throughout follicle growth, until the LH surge, which triggers the closing of gap junctions and loss of direct communication (Norris et al., 2009). The oocyte nucleus remains arrested at prophase of meiosis I until the LH surge and the closing of gap junctions. This loss of communication triggers changes in oocyte intracellular signaling and the resumption of meiosis, to arrest again at metaphase of meiosis-II (MII) (Rodrigues et al., 2008; McGinnis et al., 2018; Figure 1B).

The stockpiling and follicle growth do not occur if germ–somatic cell interaction is impaired, ultimately leading to female infertility (Li and Albertini, 2013; Clarke, 2017). For example, the GDF9 null mouse ovaries exhibit a reduction of TZPs with altered position, parallel to the oocyte rather than perpendicular, resulting in arrest of follicle development at primary stage and no ovulation (Carabatsos et al., 1998; Sanfins et al., 2018; Figures 1B, 2E,F). In human, GDF9 is essential for cumulus expansion and normal ovulation (Chang et al., 2017). Moreover, a significantly higher number of TZPs were observed in the oocyte–granulosa interface of FSHβ-deficient mice, implying FSH antagonist action toward TPZs in the zona pellucida (Combelles et al., 2004; Clarke, 2017).

## Concluding Remarks

This review highlights the importance of germ–somatic cell communication for not only primordial follicle assembly and establishment of the ovarian follicle reserve, but ultimately building a foundation for an optimal female reproductive lifespan.

Although some of these aspects are known, many more are not understood. The future for complete understanding and the possibility of creating solutions to various infertility situations will involve transcriptome analysis and research of uncovered pathways of specific signature genes. Such studies must be complemented with basic cellular research and/or gene disruption. A clear example is the recent uncovering, using RNA sequencing, of specific gene signatures and key pathways for human oocytes and the surrounding granulosa cells. Among these were five different pathways involved in primordial follicle activation, which, if investigated, may shed some light in the treatment of women with POI (Zhang et al., 2018).

## Author Contributions

PR, DL, LM, and CP contributed to writing of the manuscript. MM and JA contributed to editing and discussion. All authors contributed to the article and approved the submitted version.

## Conflict of Interest

The authors declare that the research was conducted in the absence of any commercial or financial relationships that could be construed as a potential conflict of interest.
